# Residential greenness and birth outcomes: evidence for reduced low birth weight from an umbrella review and harmonized normalized difference vegetation index synthesis

**DOI:** 10.3389/fpubh.2026.1816767

**Published:** 2026-06-18

**Authors:** Lisa Bauleo, Lucia Fazzo, Ivano Iavarone, Roberto Pasetto, Amerigo Zona

**Affiliations:** Department of Environment and Health, Italian National Institute of Health, Rome, Italy

**Keywords:** greenness, low birth weight, meta-analysis, pregnancy outcomes, preterm birth, residential exposure, urban health

## Abstract

**Introduction:**

Residential green space is increasingly recognized as a potential protective factor for maternal and neonatal health, yet the evidence regarding its association with low birth weight (LBW) and preterm birth (PTB) remains inconsistent. Existing reviews differ substantially in exposure assessment, analytical methods, and reporting standards. The objective of this umbrella review was to evaluate the strength and coherence of current evidence and to generate harmonized quantitative estimates suitable for use in environmental health assessment.

**Methods:**

Systematic reviews published from 2020 to April 2025 were identified using predefined PECOS (Population, Exposure, Comparator, Outcome, Study design) criteria. Review quality was assessed using Measurement Tool to Assess Systematic Reviews (AMSTAR 2) and its environmental health extension (AMSTAR 2-EH). When the body of evidence was judged at least limited, a harmonized meta-analysis of the primary studies included in previous reviews was conducted. Effect estimates were transformed to a common exposure metric corresponding to a 0.1-unit increase in the Normalized Difference Vegetation Index (NDVI) and pooled using random-effects models stratified by residential buffer size.

**Results:**

Nine systematic reviews met the inclusion criteria. The overall evidence was graded as limited for low birth weight and inadequate for preterm birth. The harmonized quantitative synthesis showed consistent reductions in LBW risk in association with 300 and 500 meter buffers, with the strongest and most consistent association observed for the 300 meter buffer. Evidence for PTB was heterogeneous and insufficient to support a pooled estimate.

**Discussion:**

Residential greenness is associated with reduced LBW risk, while evidence for PTB remains inadequate, reflecting inconsistent findings across reviews and important methodological limitations. Harmonized estimates provide sound parameters for health impact assessment and support the integration of green infrastructure into urban and maternal health policies.

**Systematic review registration:**

https://www.crd.york.ac.uk/PROSPERO/view/CRD42025646790, PROSPERO CRD42025646790.

## 1 Introduction

More than half of the global population currently resides in urban areas, and this figure is expected to reach almost 70% by 2050 (1). Rapid urbanization has led to increased exposure to air pollution and to a range of environmental and social stressors—including degraded physical environments, reduced access to green spaces, diminished social support networks, and limited healthcare access—that are closely linked to adverse health outcomes (2).

This global increase in urban populations is exacerbated by climate change. The urbanization contributes to environmental factors accounting for approximately 22% of the global disease burden, (3, 4).

In this context, urban green spaces have emerged as potential buffers against the negative health effects of urban living. Many studies support that exposure to green environments and urban green spaces may promote physical and mental wellbeing, mitigate exposure to heat, noise, and air pollution, and foster healthier behaviors (5–11). However, though the increasing availability of studies supports a positive health effect of green spaces, the availability and quality of green spaces within urban areas is often uneven, raising concerns about environmental health inequalities and environmental justice (12).

Environmental exposures during pregnancy have been identified as possible key determinants of maternal and neonatal health (13, 14). Low birth weight (LBW) and preterm birth (PTB) are among the most critical indicators of neonatal vulnerability and predictors of long-term complications (15, 16). According to the WHO, 99% of the global population lives in areas with poor air quality, which exacerbates health risks for pregnant women and newborns through mechanisms such as oxidative stress, inflammation, and metabolic dysregulation (17).

Epidemiological research on the association between residential greenness and pregnancy outcomes has increased in recent years. Meta-analyses of epidemiological studies (18, 19) suggest that higher Normalized Difference Vegetation Index (NDVI) values, a widely-used metric quantifying the health and density of vegetation around maternal residences, are associated with modest increases in birth weight and reduced odds of LBW and small for gestational age (SGA), while findings for PTB remain inconsistent. Islam et al. (20) highlight that green space exposure during pregnancy is associated with reduced risk of infantile atopic dermatitis and improved perinatal outcomes, with stronger effects among mothers with lower educational attainment. Moreover, smaller distance from urban parks has been linked to a reduced risk of lower birth weight and gestational age (21, 22).

Association between nature contact and children's socio-emotional development, i.e., improvements in working memory, self-regulation, play quality, and reductions in behavioral symptoms, was reported (23). The effects vary depending on urban context, type of exposure (access vs. direct interaction), and modifying factors such as gender, ethnicity, and socioeconomic status (23).

Despite growing interest, the literature remains fragmented. Original studies vary widely in design, exposure metrics (e.g., NDVI, proximity to parks), outcome definitions, and control for confounding and effect-modifying factors. Published systematic reviews and meta-analyses focus on specific outcomes, populations, or exposure definitions and few reviews have systematically addressed how contextual factors—such as socioeconomic status, urban characteristics, and climate—interact with green space exposure to influence pregnancy outcomes (12, 19).

Earlier evidence published prior to the 2020–2025 search window suggested a beneficial association between residential greenness and selected birth outcomes, particularly LBW (24–27).

Large population-based cohort studies conducted in Europe, North America, and the Middle East reported birth-weight increases ranging approximately from 20 to 45 g per interquartile range increase in the Normalized Difference Vegetation Index (NDVI), together with modest reductions in the risk of low birth weight (28, 29).

Earlier studies also highlighted variability related to exposure metrics. While NDVI emerged as the most consistently associated indicator across settings, alternative metrics such as the Enhanced Vegetation Index (EVI) and three-dimensional LiDAR-based measures were occasionally reported as equally or more predictive of fetal growth outcomes. In contrast, the indicator based on percent tree canopy often showed weaker or inconsistent associations after comprehensive adjustment (30–32).

Evidence from formal mediation analyses suggests that air pollution explains only a limited proportion of the association between greenness and birth outcomes. Several studies have examined both mediation and interaction effects, consistently indicating that the protective effects of residential greenness are only partially attributable to reduced air pollution exposure. Instead, a substantial proportion of the association appears to operate through alternative pathways, including heat mitigation, psychological stress reduction, and broader psychosocial mechanisms (33, 34).

Despite this substantial body of earlier evidence, previous reviews emphasized persistent heterogeneity in study design, exposure metrics, and analytical approaches, limiting comparability and the derivation of standardized quantitative estimates. These limitations motivated the present umbrella review, which aims to determine whether more recent evidence (2020–2025), combined with harmonized exposure metrics, improves coherence and yields estimates suitable for Health Impact Assessment (HIA).

This paper presents an umbrella review aimed at synthesizing and critically evaluating existing evidence from systematic reviews and meta-analyses on the relationship between green space exposure and LBW and PTB prevalence. In addition, this review performs a new quantitative reanalysis, applying standardized exposure metrics and rigorous quality criteria. The provided meta-analytical estimates of the benefit linked to greenness on adverse reproductive outcomes could inform HIA practice, a framework used to evaluate the potential health implications of policies or interventions by combining exposure–response functions with population data. These methodological enhancements aim to reduce heterogeneity, improve comparability, and provide a robust basis for future research and urban health policy (35).

## 2 Materials and methods

The methods used for conducting the umbrella review are those recommended by Belbasis et al. (36), applying the Preferred Reporting Items for Systematic Reviews and Meta-analyses (PRISMA) reporting standards (37). The protocol of this umbrella review was registered in the International Prospective Register of Systematic Reviews - PROSPERO (Registration no. CRD42025646790).

### 2.1 Eligibility criteria

The eligibility criteria were defined using the PECOS framework (Population, Exposure, Comparator, Outcome, Study design), as summarized:

- Population: pregnant individuals or neonates.
- Exposure: residential exposure to green space, assessed through objective or validated measures such as NDVI, proximity to parks, tree canopy coverage, or other land-use classifications. Studies using subjective measures of greenness were excluded unless supported by spatial data.
- Comparator: no restrictions were applied regarding comparators; studies with or without control groups were considered.
- Outcomes: low birth weight (LBW) and preterm birth (PTB). Studies reporting small for gestational age (SGA), gestational age (GA), or other perinatal indicators were considered if they also included LBW or PTB.
- Study design: only systematic reviews were included. Reviews had to report a structured methodology, including a search strategy and eligibility criteria. Narrative reviews, commentaries, and scoping reviews were excluded.

Exclusion criteria included discrepancies with the predefined PECOS framework, such as differences in the target population, exposure definition (e.g., non-residential or subjective greenness measures), outcomes other than low birth weight or preterm birth, or study designs not meeting the criteria for a systematic review.

### 2.2 Data sources and search strategy

The literature search was conducted in PubMed, EMBASE and Scopus.

PubMed includes MEDLINE-indexed articles as well as additional records from PubMed Central and other sources. To ensure consistency and methodological rigor, the initial search strategy was developed in PubMed using a combination of MeSH terms (Medical Subject Headings) and free-text keywords. The strategy was then adapted for EMBASE and Scopus using the SR Accelerator Polyglot Search Translator, a validated tool that facilitates accurate translation of search strings across databases. Full details of the search strategies are provided in the Table S1. Only reviews published in English or Italian were considered. The search targeted systematic reviews and meta-analyses published between January 2020 and 22 April 2025 (date of the search). The decision to focus on the most recent five-year period reflects the rapid expansion of research in this field, the emergence of refined exposure metrics—such as high-resolution NDVI and stratified buffer analyses—and the increasing adoption of suitable standardized methodological frameworks in environmental epidemiology and urban health research. This time frame ensures that the included studies reflect the most current scientific evidence and are aligned with contemporary standards.

### 2.3 Literature screening and study selection

Following deduplication using Endnote, each study/record was screened independently by two panels of reviewers (each consisting of two researchers, who worked in blind: panel 1 L.B. and L.F; panel 2 I.I., A.Z.). Screening was done in a first step by reading the title and abstract, and in a second step based on the full-text analysis.

Each panel applied the criteria separately to ensure methodological rigor and reduce selection bias. The final reconciliation of included and excluded studies was performed by the entire reviewers' panel. The complete list of screened studies, including bibliographic details, inclusion decisions (at title/abstract and full-text level), and reasons for inclusion or exclusion, is reported in Table 1. This table was compiled and verified independently by two researchers to ensure consistency and transparency in study selection. Finally, all the selected articles were retained for data extraction.

**Table 1:** Study selection process and inclusion/exclusion decisions at title/abstract and full-text screening.

Ref	Author	Year	Title	Source	DOI	Included - title or abstract	Included - full text	Reason for inclusion	Reason for exclusion
1	Ahmer Z	2024	Association between residential green spaces and pregnancy outcomes: a systematic review and meta-analysis	Int J Environ Health Res. (2024) 7:1–18	10.1080/09603123.2023.2299242	Yes		Review of residential green spaces and pregnancy outcomes with meta-analyses	
2	Rahimi-Ardabili H	2021	Green Space and Health in Mainland China: A Systematic Review	Int J Environ Res Public Health. (2021) 18:9937	10.3390/ijerph18189937	See full text	No		There was insufficient evidence to draw firm conclusions on mortality, birth outcomes
3	Rigolon A	2021	Green Space and Health Equity: A Systematic Review on the Potential of Green Space to Reduce Health Disparities	Int J Environ Res Public Health. (2021) 18:2563	10.3390/ijerph18052563	See full text	Yes	In this review on Green Space and Health Equity, Birth outcomes (PTB, LBW, etc.) were included in all the search strategies.	
4	Hu CY	2021	Residential greenness and birth outcomes: A systematic review and meta-analysis of observational studies	Environ Res. (2021) 193:110599	10.1016/j.envres.2020.110599	Yes		Review of green areas with meta-analytic measures for preterm birth and low birth weight	
5	Mygind L	2021	Landscapes of becoming social: A systematic review of evidence for associations and pathways between interactions with nature and socioemotional development in children	Environ Int. (2021) 146:106238	10.1016/j.envint.2020.106238	See full text	Yes	The review include exposure and outcome under study	
6	Lee KJ	2020	Greenness, civil environment, and pregnancy outcomes: perspectives with a systematic review and meta-analysis	Environ Health. (2020) 19:91	10.1186/s12940-020-00649-z	See full text	Yes	Review of green areas with meta-analytic measures for preterm birth and low birth weight	
7	Wolf KL	2020	Urban Trees and Human Health: A Scoping Review	Int J Environ Res Public Health. (2020) 17:4371	10.3390/ijerph17124371	No			Not a systematic review (scoping review)
8	Akaraci S	2020	A Systematic Review and Meta-Analysis of Associations between Green and Blue Spaces and Birth Outcomes	Int J Environ Res Public Health. (2020) 17:2949	10.3390/ijerph17082949	See full text	Yes	Review of green and blue areas with meta-analytic measures for birth weight, preterm birth, and low birth weight	
9	Zhan Y	2020	Influence of residential greenness on adverse pregnancy outcomes: A systematic review and dose-response meta-analysis	Sci Total Environ. (2020) 718:137420	10.1016/j.scitotenv.2020.137420	Yes		To estimate the association and dose-response relationship between residential greenness and pregnancy outcomes	
10	Islam	2020	Green space and early childhood development: a systematic review	Rev Environ Health. (2020) 35:189–200.	10.1515/reveh-2019-0046	Yes		Systematic review. Among the various outcomes, the effect of green exposure during pregnancy on low birth weight (LBW) is studied.	
11	Mannucci	2023	Air pollution, cardiovascular disease, and urban greening: an ecological blueprint	Eur J Prev Cardiol. (2023) 30:1608–11.	10.1093/eurjpc/zwad119	See full text	No		Not a systematic review (narrative review)
12	Mavoa	2021	Parental Preconception Exposures to Outdoor Neighborhood Environments and Adverse Birth Outcomes: A Protocol for a Scoping Review and Evidence Map	Int J Environ Res Public Health. (2021) 18:8943.	10.3390/ijerph18178943	No			Not a systematic review (scoping review)
13	Schutte	2021	Hypertension in Low- and Middle-Income Countries	Circ Res. (2021) 128:808–26.	10.1161/CIRCRESAHA.120.318729	No			Not a systematic review (compendium)
14	Siddika	2023	The impact of place-based contextual social and environmental determinants on preterm birth: A systematic review of the empirical evidence	Health Place. (2023) 83:103082.	10.1016/j.healthplace.2023.103082	Yes		Systematic review. Among the various outcomes, the effect of green exposure during pregnancy on preterm birth (PTB) is studied.	
15	Singh	2020	Prevalence and Social and Built Environmental Determinants of Maternal Prepregnancy Obesity in 68 Major Metropolitan Cities of the United States, 2013-2016	J Environ Public Health. (2021) 2021:6650956.	10.1155/2021/6650956	No			Research study. Preterm birth is cited, related to maternal obesity.
16	Wies	2024	Urban environment and children's health: An umbrella review of exposure response functions for health impact assessment	Environ Res. (2024) 263:120084.	10.1016/j.envres.2024.120084	See full text	No		Only one review included in this umbrella review analyze che relation beetween green exposure and birth outcome and this review has already been included

### 2.4 Data collection process and data items

The entire team of reviewers carried out the data extraction for the selected articles, with each article independently analyzed by the two panels. Information on study design, population characteristics, exposure assessment and measurement methods, statistical analyses, and funding sources was collected.

### 2.5 Quality and risk of bias assessment

The quality assessment of each selected article was performed independently by two panels, and in case of disagreement, the whole team of reviewers was consulted to reach a final agreement. To assess the quality of the systematic reviews, we followed the Measurement Tool to Assess Systematic Reviews (AMSTAR 2), proposed by Shea et al. (38). This tool counts 16 items, seven of them focusing on critical points that could affect the validity of the review, i.e., the registration of the protocol before the commencement of the review, adequacy of the literature search, assessment of the risk of bias from individual studies included in the review and its consideration in the interpretation of the results, and appropriateness of meta-analytical methods (38).

The overall confidence in the results of the review was rated in four classes: “high” (no or one “non-critical” item not satisfied), “moderate” (more than one non-critical item not satisfied), “low” (one critical item not satisfied), “critically low” (more than one critical item not satisfied) (https://amstar.ca/Amstar_Checklist.php). For reviews that incorporated a meta-analysis, we additionally applied AMSTAR2-EH, an adaptation of AMSTAR 2 specifically designed for environmental health studies. AMSTAR2-EH expands the original framework by introducing criteria relevant to environmental epidemiology, such as harmonization of exposure metrics, transformation of effect estimates into a common unit, and explicit consideration of environmental confounders (21 items in total, 5 of which are critical). Under AMSTAR2-EH criteria, reviews were deemed “acceptable” only if all critical items were satisfied and no more than four non-critical items were missing; otherwise, they were classified as not “acceptable” (39).

### 2.6 Evaluation of strength of the body of evidence

The strength of the evidence of association between each birth outcome and green exposure was evaluated considering the reliability of each systematic review — defined in terms of review quality, transparency, and internal consistency — together with the concordance of findings across reviews. The body of the evidence was evaluated separately for the two outcomes.

The evidence was rated in three grades: Sufficient, Limited, Inadequate, partly derived from the approach used by the International Agency for Research on Cancer (IARC) Monograph (40), but specifically defined, as reported in Table 2.

**Table 2:** Criteria used to rate the evidence of the association between the pregnancy outcome and the greenness exposure.

Grading of the evidence	Criteria
**SUFFICIENT**: the evidence is sufficient to infer the presence of a causal association. The strength of association, considerations of dose-response relationship, temporal coherence and biological plausibility further support causality. Alternative explanations, in particular the role of random variability, bias, and confounding factors, can be reasonably excluded.	•**at least one** “high” quality systematic review, with or without meta-analyses, reports a positive and consistent association.
**LIMITED**: the evidence is limited, and not sufficient to infer the presence of a causal association. ^*^ The role of random variability, bias and confounding factors may not be completely excluded.	•**more than one** “moderate” quality systematic review, with or without meta-analysis, reports a positive and consistent association•**At least one** “moderate” quality systematic review, with or without meta-analysis, reports a positive and consistent association, and no conflicting evidence from “low” quality reviews.•**more than one** “low” quality systematic review, with or without meta-analyses, reports a positive and consistent association
**INADEQUATE**: the evidence is not adequate to infer the presence or the absence of a causal association. ^*^	•**more than one** “moderate” quality systematic review, with or without meta- analyses, investigates the association, but the results are not consistent •**more than one** “low” quality” systematic review, with or without meta-analyses, investigate the association, but the results are not consistent •**one or more than one** systematic review, with or without meta- analyses, report positive findings, but the quality has been evaluated as “critically low.”
^*^Among the concurring different risk estimates, those from higher quality studies were given higher weight.	

Only outcomes for which the overall body of evidence was assessed as at least limited were considered eligible for quantitative synthesis in the main manuscript. As the evidence for preterm birth (PTB) was graded as inadequate, due to substantial heterogeneity and inconsistent findings across the included systematic reviews, PTB was not included in the main meta-analytical assessment.

The choice of an IARC-inspired framework, rather than the GRADE approach (41), was motivated by the characteristics of the evidence on residential greenness and birth outcomes, which is predominantly observational and methodologically heterogeneous. In this context, causal interpretation relies primarily on consistency of findings, exposure–response coherence, and biological plausibility, as outlined in the IARC Monographs Preamble (38), rather than on experimental study designs.

The adopted framework does not replicate the IARC classification verbatim, but operationalizes its principles through explicitly defined criteria tailored to this umbrella review.

Reviews rated as moderate quality were given greater weight than those rated as low or critically low. AMSTAR2-EH criteria—particularly those related to exposure harmonization, transformation of effect estimates into a common metric, and risk-of-bias assessment—were used to evaluate the suitability of the evidence for quantitative synthesis, following methodological guidance for environmental health assessment). This structured integration allowed a systematic transition from review-level quality assessment to outcome-specific judgments of the overall strength of evidence.

### 2.7 Meta-analytical assessment

In addition to the narrative synthesis of the systematic reviews, a quantitative reanalysis of the primary studies was conducted, adopting a rigorous approach inspired by the methodology proposed by Forastiere et al. (39) or the selection and validation of quantitative exposure-response relationships (often termed Concentration–Response Functions, CRFs) used in health risk assessments (HRA). Since most of the included reviews did not meet the methodological quality criteria defined by the AMSTAR2-EH tool, the meta-analytic estimates reported in those reviews were not used directly. Instead, data were extracted from the original studies to produce new meta-analytical estimates. This approach was adopted only for the outcomes for which the grade of evidence was assessed as at least limited.

For each study, the effect estimates (β or OR) associated with green space exposure were identified and, when necessary, transformed into a common metric. The most reliable exposure metric was the NDVI, as it is continuous, standardized, and widely used. Estimates were harmonized to reflect an increment of 0.1 NDVI units, ensuring comparability across studies, by inverting the relationship described in Equation 1 and following the methodology described in Bauleo et al. (42). Studies using non-transformable metrics (e.g., distance to parks) were excluded from the meta-analysis.

$$\begin{array}{l} OR_{0.1}=exp\;\left(\frac{\ln\left(OR\right)}{\Delta }*0.1\right) \end{array}$$ (1)

Standardized estimates were pooled using random-effects models (REML), stratified by exposure buffer (e.g., 100 m, 300 m, 500 m, 1,000 m) to assess spatial heterogeneity. The I^2^ statistic and 95% confidence intervals were calculated. In line with Forastiere's recommendations (39), the applicability of the estimates for HIA is restricted to the exposure ranges observed in the original studies, avoiding extrapolations beyond the upper limits.

## 3 Results

**Figure 1:**
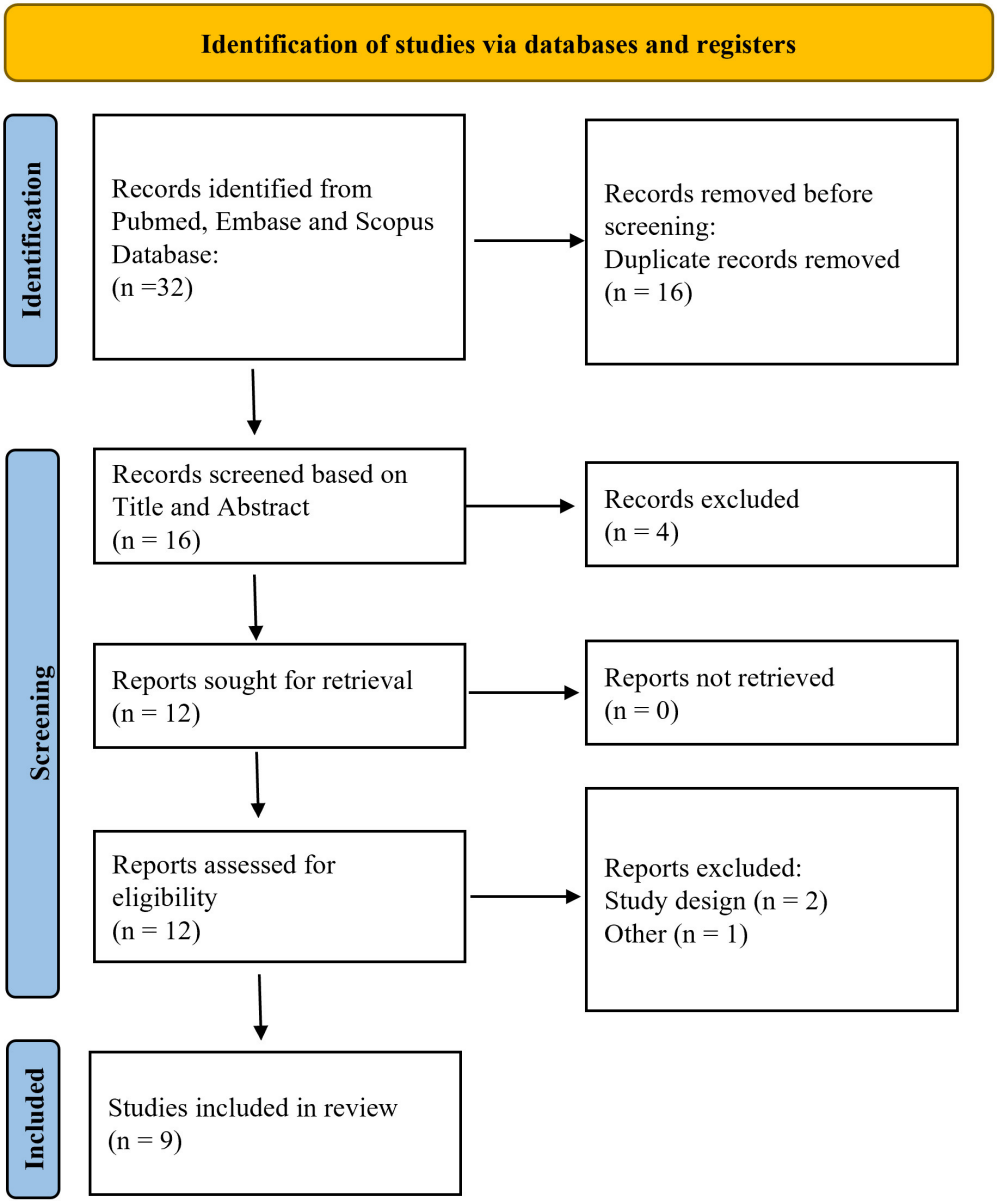
PRISMA 2020 flow diagram for systematic reviews published in the period 2020-2025, which included searches of databases and registers only. Flow diagram illustrating the identification, screening, eligibility assessment, and inclusion of systematic reviews. A total of 32 records were identified, 16 were screened after removing duplicates, 7 were excluded, and 9 systematic reviews met all eligibility criteria and were included in the umbrella review.

The study selection process is illustrated in Figure 1, following the PRISMA 2020 guidelines. A total of 32 records were identified through database searches (PubMed, Embase, Scopus) in the period 2020–2025. After removing 16 duplicates, 16 records were screened. Out of them, 4 were excluded based on title and abstract, and 3 based on the full-text analysis. Table 1 describes the included and excluded articles and the rationale. Ultimately, 9 systematic reviews were included in the umbrella review because they fit the PECOS question. Among these 9 systematic reviews, five were incorporated in the meta-analyses of primary studies (18, 19, 43–45).

### 3.1 Characteristics of included systematic reviews

**Figure 2:**
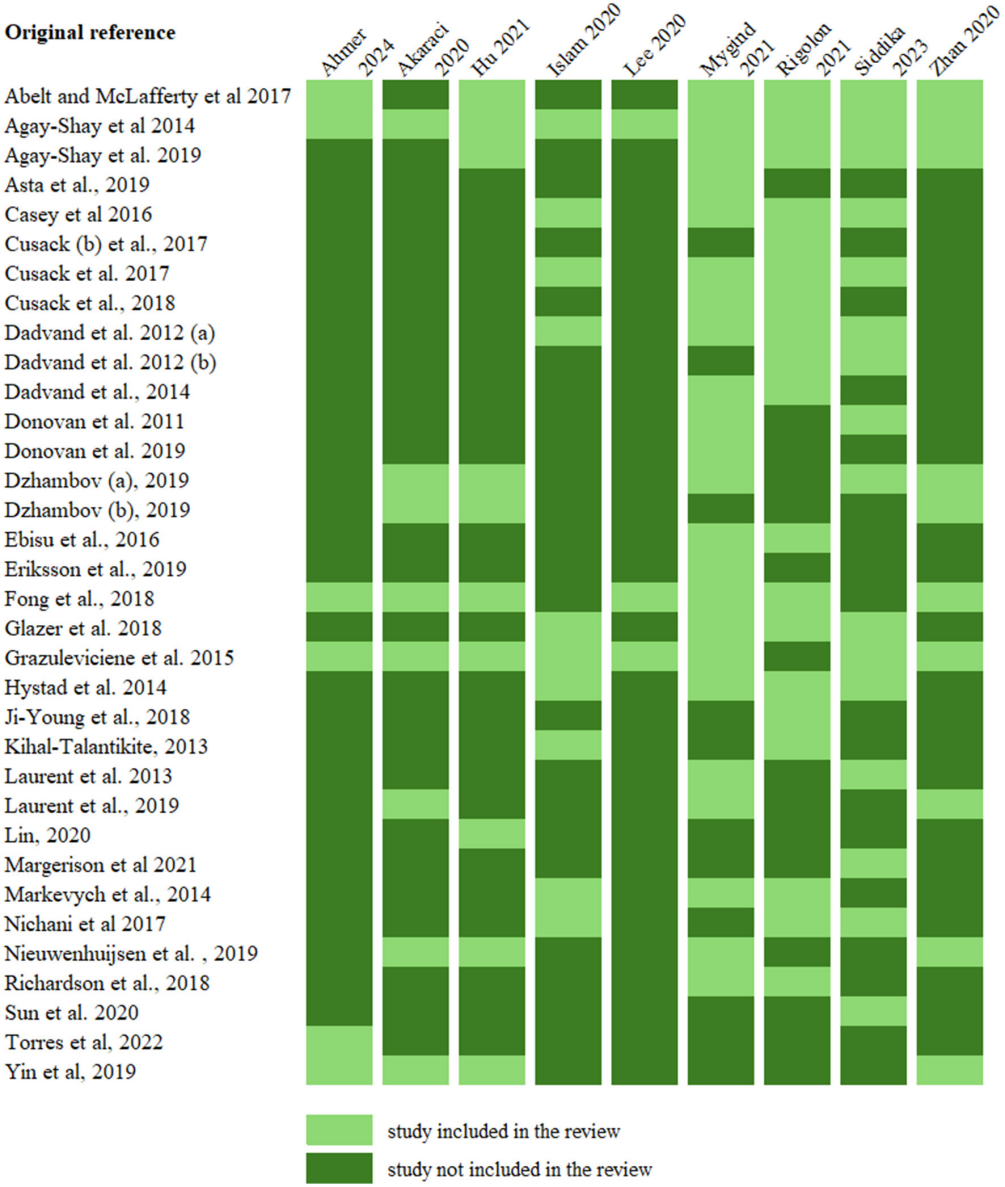
Heatmap of original studies included across the selected systematic reviews. Heatmap showing the distribution of primary studies included in the nine systematic reviews. Cross-sectional studies predominated, followed by cohort studies and fewer case-control or quasi-experimental designs.

The summary of the 9 systematic reviews included in this umbrella review is provided in Table 3. The articles were published between 2020 and 2024, five in 2020 (19, 20, 43, 44), three in 2021 (12, 18, 23), one in 2023 (46), and one in 2024 (45). All the reviews recruited primary studies conducted in multiple countries across different regions, primarily in high-income countries (North America and Europe), with some from Asia and Oceania. The 9 systematic reviews included a total of 34 distinct primary articles with a moderate overlap of 26%. Figure 2 reports the heatmap of the original studies included in the selected reviews. Cross-sectional designs predominated, followed by cohort studies and, less frequently, case-control or quasi-experimental designs. Sample sizes ranged widely, from a few hundred participants to over three million.

**Table 3:** Summary of the systematic reviews included.

Study, Country (bibliographic reference)	Literature search (sources and date)	The characteristics of included studies	Exposure measures	Studied outcomes	Statistical analysis/assessment of evidence (methods and results)	Sources of funding
Akaraci S, Feng X, Suesse T, Jalaludin B, Astell-Burt T. A systematic review and meta-analysis of associations between green and blue spaces and birth outcomes. *Int J Environ Res Public Health*. (2020) 17:2949. https://doi.org/10.3390/ijerph17082949. The studies were conducted in high-income Western countries, except for the four studies from Israel and China.	Sources Used: Science Direct, PubMed, ProQuest, Scopus, Google Scholar and reference lists of relevant publications. Date: November and December 2018	37 studies; mostly cross-sectional with some cohort, case-control, ecological, quasi-experimental; mainly high-income countries.	residential greenness NDVI within multiple circular buffer distances (50–2,000 mt) around the residential addressor centroid of residentila area	BW (continuous), LBW, PTB, SGA from official birth records.	Random-effects meta-analyses for birth weight, LBW, and PTB. Effects reported as β for continuous outcomes and ORs for categorical outcomes. High heterogeneity (I^2^ 54–86%). Findings: higher birth weight associated with greater greenness; non-significant associations for LBW and PTB. Limited evidence for blue space.	Funded by Hort Innovation with co-investment from University of Wollongong (Faculty of Social Sciences), UOW Global Challenges Initiative and Australian Government.
Hu CY, Yang XJ, Gui SY, Ding K, Huang K, Fang Y, et al. Residential greenness and birth outcomes: a systematic review and meta-analysis of observational studies. *Environ Res*. (2021) 193:110599. https://doi.org/10.1016/j.envres.2020.110599. All studies were conducted in North America or Western Europe, except for one from Israel and one from Lithuania.	Sources Used: EMBASE, Web of Science, PubMed. Date: July 10, 2020 (1 study that was unpublished at the time of the last PubMed search but has since been published was included in the analysis).	29 studies: 6 cohort, 22 cross-sectional, 1 case-control; moderate/high quality; inclusion required NDVI at maternal residence. [Table 3 -...included | Excel]	NDVI; effects harmonized per 0.1 NDVI increment and high vs. low exposure categories.	BW (continuous), LBW, PTB, SGA from clinical assessment, registries or medical records.	Random- or fixed-effects meta-analyses depending on heterogeneity. Effect estimates harmonized to 0.1-unit NDVI increments. Results: higher birth weight (≈8–15 g per 0.1 NDVI) and reduced LBW risk; no significant association with PTB. Risk of bias assessed using NTP/OHAT. Overall evidence rated as “moderate certainty.”	Supported by Anhui Medical University, Anhui Provincial Natural Science Foundation, and National Natural Science Foundation of China.
Lee KJ, Moon H, Yun HR, Park EL, Park AR, Choi H, et al. Greenness, civil environment, and pregnancy outcomes: perspectives with a systematic review and meta-analysis. *Environ Health*. (2020) 19:91 The studies were conducted in North America and Western Europe except for 5 from Israel, Lithuania, New Zeland and Malaysia.	Sources Used: MEDLINE, PubMed, EMBASE, Cochrane Library, Korean Studies Information Service System (K-eArtciles), CINHAl. Furthermore, the re lists of the relevant articles were manually searched to identify additional studies. Date: April 2019	10 NDVI-based studies; Landsat ETM+ (30 m); exclusions: non-original/irrelevant outcomes.	NDVI within buffers of 100, 250, and 500 m.	BW, LBW, VLBW, SGA, PTB/VPTB (standard definitions).	Random-effects meta-analyses stratified by NDVI buffer size, plus meta-regression to assess dose–response. Findings: increased birth weight; reduced LBW, VLBW, and SGA (pooled OR≈0.94). Small reduction in PTB for the 100-m buffer (OR 0.98). Very high heterogeneity. Risk of bias evaluated with RoBANS.	None reported.
Ahmer Z, Atif M, Zaheer S, Adil O, Shaikh S, Shafique K. Association between residential green spaces and pregnancy outcomes: a systematic review and meta-analysis. *Int J Environ Health Res*. (2024) 7:1–18. https://doi.org/10.1080/09603123.2023.2299242. USA, Canada, Spain, UK, Austria, Italy, Sweden, Lithuania, Germany, New Zealand, Scotland, Iran, China and Israel.	Sources Used: Medline/PubMed, EMBASE, Science Direct, and Google Bibliography of recent and relevant systematic reviews/meta-analyses and references cited in the relevant original articles were also checked for studies not retrieved during the independent searches Date: June 2023	31 studies: 26 cross-sectional, 3 cohort, 1 case-control, 1 quasi-experimental.	NDVI (primary); also tree canopy, LiDAR, and green space land-use; NDVI most common (n=23).	BW, LBW, PTB, SGA (sources for outcomes not consistently stated).	Random-effects meta-analyses for birth weight and LBW at NDVI buffers of 250–500 m. Findings: increased birth weight (β≈9–13 g) and reduced LBW risk (OR 0.97). Subgroup analyses by buffer size. PTB not meta-analyzed due to inconsistent exposure metrics. Substantial heterogeneity (I^2^ 70–90%).	Authors reported no funding.
Rigolon A, Browning MHEM, McAnirlin O, Yoon HV. Green space and health equity: a systematic review on the potential of green space to reduce health disparities. *Int J Environ Res Public Health*. (2021) 18:2563. https://doi.org/10.3390/ijerph18052563. All continents, except Africa. The results mainly refer to North America (mainly US) and Europe.	Sources Used: CINAHL, Cochrane, PubMed, Scopus, and Web of Science Additionally, they selected keywords from previous systematic reviews on green space and health and access to green space . Date: 17 April 2019	No experimental studies; mostly cross-sectional (≈88%), few longitudinal (≈12%).	Objective (GIS/NDVI, buffers or containers) and subjective measures (surveys); varied spatial resolutions (mostly 2–30 m).	Multiple health outcomes including birth outcomes (LBW, PTB, SGA; 18 papers addressing birth outcomes).	Non-meta-analytic synthesis. Methodological quality scored across four domains (study design, exposure assessment, confounder control, statistical analysis). Effect-modification analyses for SES and ethnicity using Kruskal–Wallis and Dunn tests. Findings: heterogeneous evidence; stronger greenness benefits for low-SES populations. For birth outcomes, associations were limited and inconsistent.	No external funding.
Mygind L, Kurtzhals M, Nowell C, Melby PS, Stevenson MP, Nieuwenhuijsen M, et al. (2021). Landscapes of becoming social: a systematic review of evidence for associations and pathways between interactions with nature and socioemotional development in children. Deakin University. Journal contribution. https://hdl.handle.net/10536/DRO/DU:30145510 22 studies out of 43 selected stemmed from Europe, 12 from North America, 4 from Australasia, 2 from South Korea and one from Israel and Iran.	Sources Used: Embase, Environment Complete, MEDLINE, and APA PsycINFO. The scoping review approach was based on a snowball method using the seminal publication by Hartig et al. (67) as a so-called seed publication. The seed paper is a review of reviews exploring benefits of interaction with natural environments on mental, physical, and social health outcomes. The publication had been cited more than 460 times on the 21st of May 2019, when the search was commenced, making it one of the most cited articles in the field. Date: September 2019	43 studies on socioemotional outcomes (26 observational, 17 experimental) selected from 223; < 12 years; multiple regions.	Availability/access or interaction with nature; operationalized via NDVI, distance to green space, presence/absence, and land-use classifications.	Socioemotional domains and proximal outcomes; birth outcomes included among proximal domains.	Narrative synthesis (no meta-analysis). Counted positive/negative/non-significant associations and evaluated risk of bias; p-curve analysis conducted when sufficient data available. Findings for birth outcomes: small, inconsistent effects with substantial methodological heterogeneity and frequent risk of bias.	Scholarships/awards (e.g., Deakin University; ARC Future Fellowship for a co-author).
Zhan Y, Liu J, Lu Z, Yue H, Zhang J, Jiang Y. Influence of residential greenness on adverse pregnancy outcomes: a systematic review and dose-response meta-analysis. *Sci Total Environ*. (2020) 718:137420. https://doi.org/10.1016/j.scitotenv.2020.137420. Almost half of the studies (*n* = 17) were conducted in Americas, 12 in Europe, 5 in Asia and 2 in Oceania	Sources Used: PubMed, Embase, Ovid, Scopus and Web of Science Date:1 December, 2019	36 studies were included: 14 cohort studies, 2 case-control studies and 19 cross-sectional	NDVI (continuous/categorical), proximity/distance to green space, percentage of green space, tree canopy; buffers 100–1,000 m.	BW, head circumference, gestational age (continuous); LBW, PTB, SGA, GDM, GH, preeclampsia, mental disorders (categorical).	Random-effects meta-analyses and linear dose–response models. Effects reported as β and ORs. Findings: consistently higher birth weight across all NDVI buffers (100–1,000 m) and reduced LBW risk (OR 0.82–0.90). No significant association with PTB. Dose–response: 2% reduction in LBW risk per 0.1 NDVI at 300 m. Very high heterogeneity (I^2^>70%).	Supported by CAMS Initiative for Innovative Medicine (grant 2019-I2M-2-007).
Islam MZ, Johnston J, Sly PD. Green space and early childhood development: a systematic review. *Rev Environ Health*. (2020) 35:189–200. https://doi.org/10.1515/reveh-2019-0046 Eleven of the studies identified in this review focused on the association between maternal green space exposure and perinatal health. Among these, three are from the USA and one each from Israel, Spain, Lithuania, Canada, Germany, France, Scotland and New Zealand.	Sources Used: PubMed, MEDLINE, Web of Science, MeSH and PsycINFO. Additional papers were identified by scanning the reference list of appropriate studies. Date:	23 studies (11 perinatal); perinatal subset: 3 cohort, 8 cross-sectional; often large registry-based samples.	NDVI around maternal residence; exposure ascertainment sometimes not clearly stated.	Perinatal outcomes: BW, LBW, PTB.	Narrative synthesis (no meta-analysis). Findings: increased birth weight and reduced LBW/VLBW associated with greenness; PTB results mixed and sensitive to SES and environmental confounders. Many included studies had substantial residual confounding.	Authors state no funding involved.
Siddika N, Song S, Margerison CE, Kramer MR, Luo Z. The impact of place-based contextual social and environmental determinants on preterm birth: a systematic review of the empirical evidence. *Health Place*. (2023) 83:103082. https://doi.org/10.1016/j.healthplace.2023.103082. 17 of the studies identified in this review focused on grenness and preterm birth. Among these, 8 are from the USA (New York cities, Pennsylvania, Texas, Oregon, Rhode Island, California, Michigan) two from Israel and Spain, and one each from Lithuania, Austria/Italy, Canada, and New Zealand.	Sources Used: PubMed, Embase, and CINAHL Date: mid-August 2021	41 studies total; 17 examined greenness and PTB (1 cohort, 15 cross-sectional, 1 mixed); *n* = 1,280–4,385,997.	Fourteen of the total seventeen studies measured greenness by the normalized difference vegetation index (NDVI) with various buffer sizes (from 50 m to 2,000 m) around maternal addresses. Three studies measured the percentage of green space, tree canopy (using Metro land-cover classification 2007), and green cover (using Vegetation Continuous Fields (VCF) in different census area units and in the buffers. Four of the studies also measured greenness as a binary variable based on proximity to major green spaces within a buffer of 300 m or 500 m from the boundaries of a major green space to the maternal home address.	PTB (mostly < 37 weeks), with subcategories for moderate/very PTB; data largely from vital registries; few studies distinguished spontaneous vs. indicated PTB.	Individual studies primarily used logistic regression; no pooled meta-analysis. Qualitative synthesis of findings: PTB associations highly heterogeneous; about half null and half protective (OR 0.78–0.98). One study reported increased PTB risk after advanced adjustment. Studies using alternative greenness metrics also produced inconsistent results. Quality assessment (NIH): 12 of 17 studies rated adequate.	Supported by Michigan State University Discretionary Funding Initiative.

Additional information on the socio-geographical context, environmental settings, and key effect modifiers described in the included systematic reviews is provided in Table S2. This contextual information is important for interpreting heterogeneity and for the potential application of exposure–response estimates in HIA, where interactions between greenness and other environmental and social determinants—such as air pollution, temperature, seasonality, and socioeconomic conditions—are increasingly recognized as critical.

Several of the synthesized meta-analyses explicitly adjusted for temporal and environmental covariates, including seasonality and temperature, and highlighted the role of socioeconomic factors as potential effect modifiers. For example, Hu et al. (18) reported consistent positive associations between NDVI and birth weight, with increases up to 15.35 g per 0.1-unit increment, along with reduced odds of low birth weight (OR up to 0.79), supporting the relevance of multi-stressor approaches for HIA.

#### 3.1.1 Greenspace exposure measures

Across the 9 systematic reviews, residential greenness was most frequently assessed using the Normalized Difference Vegetation Index. NDVI was typically calculated within circular buffers around maternal residences, ranging from 50 m to 1,000 m, and occasionally up to 2,000 m (18, 19, 43–45). Several reviews also reported alternative or complementary indicators, including:

- Proximity to parks or major green spaces (binary or continuous measures) (12, 20, 46).
- Percentage of green space cover based on land-use classifications (44, 45).
- Tree canopy coverage derived from aerial imagery (45).
- Advanced remote sensing techniques such as LiDAR (Light Detection and Ranging)^1^ in isolated cases (45).
- Access and interaction with natural environments, including presence/absence of green spaces and municipal classifications (23).

Buffer size selection varied substantially across studies, with 100 m, 250 m, and 500 m being the most frequently analyzed. Some meta-analyses explored dose–response relationships, harmonizing effect estimates per 0.1 NDVI unit increase (18, 44).

#### 3.1.2 Health outcomes

The primary outcomes assessed across the included reviews were low birth weight (LBW) and preterm birth (PTB), with several reviews also considering related indicators such as birth weight (BW) as a continuous measure and small for gestational age (SGA) (18–20, 43–46). Outcome definitions were generally standardized, with LBW defined as < 2,500 g and PTB as delivery before 37 weeks of gestation. Data sources were primarily birth registries, medical records, or vital statistics, ensuring comparability across studies. While most reviews focused on perinatal outcomes, (12) examined birth outcomes within a broader equity framework, exploring effect modification by socioeconomic status and race/ethnicity. Mygind et al. (23) included birth outcomes as part of proximal socio-emotional development indicators, though these were not the main focus of the review.

### 3.2 Quality and risk of bias assessment

**Figure 3:**
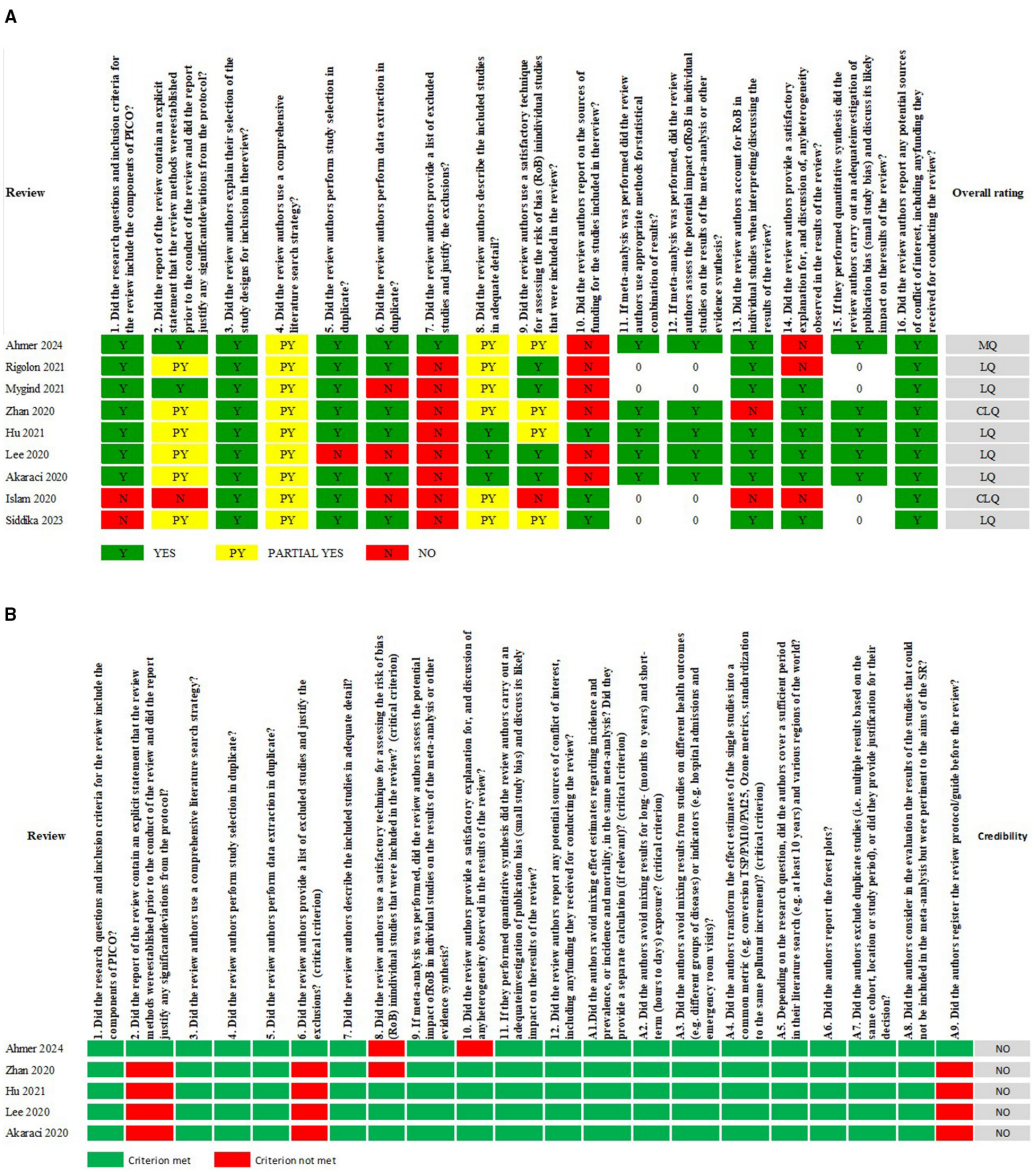
Quality assessment of the included systematic reviews using AMSTAR 2 **(A)** and AMSTAR2-EH **(B)**. **(A)** AMSTAR 2 ratings for the nine systematic reviews included in the umbrella review. One review was rated as moderate quality, six were rated as low quality, and two as critically low quality. The most frequent shortcomings involved inadequate reporting of excluded studies, insufficient assessment of risk of bias in included studies, and high heterogeneity in exposure and outcome definitions. **(B)** Quality assessment of the five reviews including a meta-analysis, evaluated using AMSTAR2-EH. None of the reviews met the criteria for an acceptable rating, which requires satisfying all critical items and missing no more than four non-critical items. Frequent limitations included lack of harmonization of exposure metrics, incomplete risk-of-bias assessment, and insufficient justification of excluded studies.

According to AMSTAR 2 tool, only one review was rated as moderate quality (45), six reviews were rated as low (12, 18, 19, 23, 43, 46), and two were rated as critically low quality (20, 44). These lower ratings were attributed to limitations in study design, potential publication bias, the methods used to combine study results, characterized by a high heterogeneity in definitions of exposure and outcome. However, reviews with greater methodological rigor often provided more reliable and consistent evidence. For the five reviews that included a meta-analysis, quality was also assessed using AMSTAR2-EH. None of these reviews achieved an acceptable rating according to the tool's criteria, which require all critical items to be satisfied and no more than four non-critical items missing. Ahmer et al. (45) failed to meet the criterion on risk-of-bias assessment of included studies using specified tools and domains, Hu et al. (18), Lee et al. (43), and Akaraci et al. (19) did not provide a list or justification of excluded studies, and Zhan et al. (44) failed to meet both critical requirements. Figures 3A, B report the detailed item-level results for both evaluations, showing for each review which AMSTAR 2 and AMSTAR2-EH items were met, partially met, or not met. This graphical representation allows readers to identify the specific methodological domains driving the overall quality ratings.

### 3.3 Evaluation of strength of the body of evidence

The included reviews consistently reported an inverse association between residential greenness and risk from adverse pregnancy outcomes, although the strength and precision from these associations varied across studies. Most reviews focused on exposure measured by NDVI within buffers of 100–500 m around maternal residences.

Meta-analyses included in five reviews generally indicated that higher NDVI values were associated with increased birth weight and reduced odds of LBW. For example, Hu et al. (18) reported that each 0.1-unit increase in NDVI was linked to a birth weight gain ranging from 7.99 g (95% CI: 4.29–11.70) to 15.35 g (95% CI: 11.41–19.29) and lower odds of LBW (OR range: 0.79–0.93). Similarly, Zhan et al. (44) found a dose–response relationship, with a 2% reduction in LBW risk per 0.1 NDVI increment (OR: 0.98; 95% CI: 0.97–0.99). Ahmer et al. (45) confirmed these findings, reporting pooled estimates for NDVI at 250 m and 500 m buffers associated with birth weight increases of 8.95 g and 12.83 g, respectively.

The results reported for PTB are less consistent. While Lee et al. (43) observed a small but statistically significant reduction in PTB risk for NDVI within 100 m (OR: 0.98; 95% CI: 0.97–0.99), other reviews, including Hu et al. (18) and Zhan et al. (44), reported null associations. Siddika et al. (46) highlighted substantial heterogeneity, with some studies suggesting beneficial effects and others reporting no association or even increased risk after adjustment for unmeasured confounders.

Overall, the grading of evidence based on quality of each study, and the consistency between the results, was defined as limited for LBW and as inadequate for PTB. While the direction of effect for LBW appears robust across reviews, high heterogeneity and methodological weaknesses reduce confidence in pooled estimates. For PTB, the evidence remains inconclusive, with mixed findings and insufficient methodological rigor to support a definitive conclusion.

### 3.4 Meta-analytical assessment

Since none of the five systematic reviews that included meta-analyses met the methodological quality criteria defined by the AMSTAR2-EH tool, a new meta-analysis limited to LBW was conducted using the original studies included in those reviews.

Only studies that assessed residential green space exposure through NDVI and allowed for the transformation of effect estimates into a harmonized metric—based on increments of 0.1 NDVI units—were selected. This approach addressed the methodological limitations of the existing reviews and ensured greater consistency in the quantitative synthesis.

**Figure 4:**
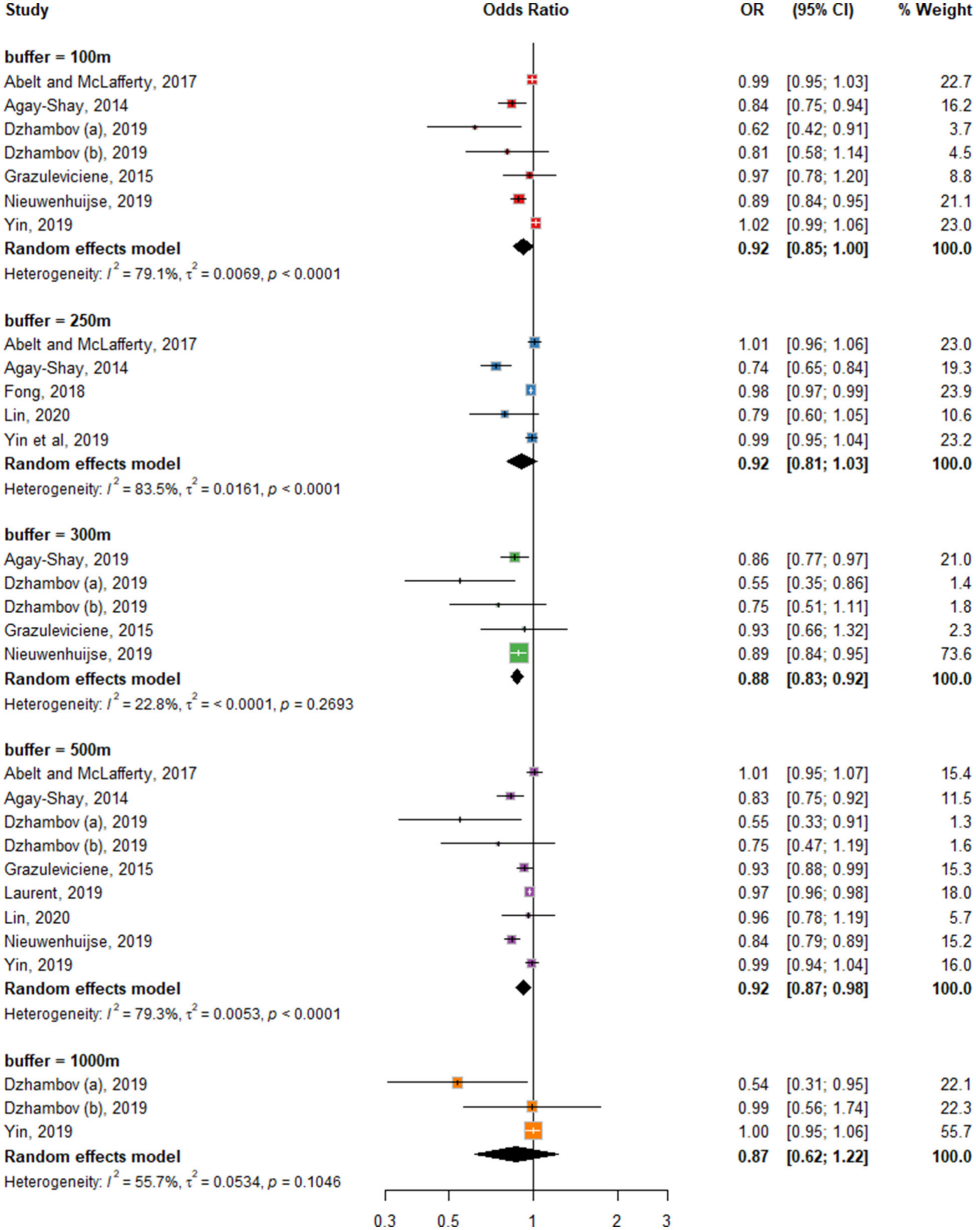
Harmonized meta-analysis of the association between residential greenness (NDVI) and low birth weight. Pooled odds ratios (OR) per 0.1-unit increase in the Normalized Difference Vegetation Index (NDVI), stratified by residential buffer size (100 m, 250 m, 300 m, 500 m, 1,000m).

The results, reported in Figure 4, indicate an inverse association between green space exposure and the risk of LBW, for all the considered buffers. Specifically, the pooled odds ratio for the 500 m buffer is statistically significant [0.92 (95% CI: 0.86–0.97)], even if the high heterogeneity (I^2^ > 75%) reflected substantial variability across studies in terms of context, population characteristics, and exposure definitions. Of particular interest is the result for the 300 m buffer, which showed a statistically significant association (OR = 0.88; 95% CI: 0.83–0.92) with low heterogeneity (I^2^ = 23%), suggesting greater consistency among the included studies despite differences in design and setting. This finding highlights the 300 m buffer as the most consistent exposure metric among those analyzed. While results for other buffers do not support the definition of a single continuous exposure–response function across different buffer sizes, they provide reliable estimates for specific, discrete distances, which can be used in impact assessment models to quantify the protective effects of green space on LBW risk.

## 4 Discussion

This umbrella review confirms a consistent inverse association between residential green space exposure and low birth weight (LBW), while evidence for preterm birth (PTB) remains inadequate.

The meta-analysis conducted in this study, based on harmonized exposure metrics from original studies, identified significant protective effects on LBW for NDVI within 300 m, and 500 m buffers, with the 300 m buffer showing the strongest and most consistent association (OR = 0.88; 95% CI: 0.83–0.92; I^2^ = 23%). These findings highlight the 300 m buffer as the most reliable metric and provide discrete estimates that may inform Health Impact Assessment (HIA) models to quantify the protective effects of green space on adverse reproductive outcomes. However, the use of these estimates for HIA should be restricted to the exposure ranges observed in the original studies and accompanied by careful consideration of environmental and social confounders to ensure epidemiological validity and robustness of inference. In the present analysis, the exposure ranges covered by the original studies predominantly reflect NDVI values observed in urban and peri-urban residential settings, spanning low to moderate–high levels of greenness within buffers between 100 and 1,000 m around maternal residences. Accordingly, the derived estimates are most relevant to scenarios involving incremental increases in residential greenness within built environments, rather than to extreme contrasts between highly urbanized and fully natural or rural contexts.

For example, in an urban area where the baseline prevalence of low birth weight is known and NDVI distributions around maternal residences are available, the pooled estimate identified for the 300 m buffer could be used to quantify the expected change in LBW cases associated with an intervention increasing average residential NDVI by 0.1 units. By combining the exposure–response estimate with population data and assuming no extrapolation beyond observed NDVI ranges, such calculations could support scenario-based HIA of urban greening policies.

Evidence for PTB remains inadequate, with inconsistent findings across reviews and substantial heterogeneity. This suggests that green space exposure alone may not substantially influence PTB risk, which is strongly shaped by socio-demographic factors such as maternal age, education level, ethnicity, and smoking habits (47). Several studies have examined interactions between greenness and other risk factors for PTB, controlling for these confounders. For example, Sun et al. (48) analyzed over three million births in California and found that green space did not strongly reduce PTB risk overall but mitigated the adverse effects of air pollution. Similarly, Mi et al. (49) reported that NDVI exposure was associated with lower PTB risk more significantly in areas with high pollutant levels, while Laurent et al. (50) observed modest protective effects after adjusting for maternal age, education, ethnicity, and smoking. Although these findings point to potential synergistic benefits, results remain heterogeneous, underscoring the need for longitudinal studies with refined exposure metrics and comprehensive adjustment for confounders.

Our findings reinforce the importance of adopting rigorous methodological approaches when assessing greenspace exposure. As highlighted by Zare Sakhvidi et al. (51), buffer selection should not be arbitrary but guided by hypothesized mechanisms, population characteristics, and data quality. The heterogeneity observed across systematic reviews included in our umbrella analysis reflects the lack of standardization in this field, with buffer choices and exposure metrics often poorly justified. Our approach—harmonizing effect estimates per 0.1 NDVI increment and stratifying analyses by multiple buffer sizes (100 m, 250 m, 300 m, 500 m, 1,000 m)—aligns with recommendations to test multiple scales and explicitly report the rationale for buffer selection. Notably, identifying the 300 m buffer as the most consistent for LBW is consistent with guidance to consider distances compatible with daily mobility and policy thresholds (e.g., the EU indicator of 300 m to a green space ≥ 5,000 m^2^) (52). Integrating these methodological principles into future research will reduce bias, improve comparability, and strengthen the applicability of evidence for health impact assessments and urban planning.

In this manuscript, the term “reliable” is used to indicate evidence that is methodologically suitable for quantitative synthesis and potential use in health impact assessment, based on review quality, consistency of findings, and harmonizability of exposure–response estimates.

Some degree of uncertainty in NDVI measurement is inherent in the use of remotely sensed indicators, owing to differences in satellite sensors, spatial resolution, seasonal variability, and atmospheric conditions. However, most of the primary studies and systematic reviews included in this umbrella review addressed these sources of variability by relying on validated satellite products, cloud-free imagery, consistent exposure windows, and clearly defined residential buffers.

Several reviews explicitly discussed these aspects and adopted harmonization strategies to improve comparability, such as standardizing effect estimates to fixed NDVI increments and stratifying analyses by buffer size, as reported by Hu et al. (18), Akaraci et al. (19), and Zhan et al. (44).

By focusing the quantitative synthesis on NDVI-based estimates harmonized to a common exposure scale, the present study reduces measurement-related heterogeneity and enhances comparability across studies, while acknowledging residual uncertainties inherent to satellite-derived greenness indicators.

Building on these considerations, a harmonized re-analysis of the original studies included in previous reviews was necessary to address several methodological limitations that prevented a consistent interpretation of the existing meta-analytic evidence. None of the previous reviews met the critical requirements of AMSTAR2-EH, particularly those related to exposure harmonization, transformation of effect estimates into a common metric, and transparent reporting of excluded studies. Additionally, earlier meta-analyses combined heterogeneous greenness indicators—such as NDVI, proximity measures, percent green cover, and tree canopy—that cannot be converted into a unified exposure scale and therefore cannot be meaningfully compared or incorporated into quantitative health impact assessments. For this reason, rather than replicating the literature search or generating new effect estimates, we relied on the primary studies already included in prior meta-analyses and applied stricter harmonization procedures. We focused exclusively on studies using NDVI, the only continuous indicator that can be standardized, and converted all effect estimates to increments of 0.1 NDVI units, stratifying results by homogeneous buffer distances. This approach enhances consistency, improves comparability across studies, and yields more interpretable estimates suitable for epidemiological assessment and potential HIA applications.

Beyond direct effects, green spaces may interact with other recognized risk factors for adverse pregnancy outcomes. Several studies have highlighted the modifying role of socioeconomic status (SES) in these associations. For instance, Asta et al. (53) found that the short-term effect of heat on PTB was stronger among women with low SES and those living near green spaces, suggesting that proximity alone does not guarantee protection. Similarly, Dadvand et al. (54) reported that the association between residential greenness and birth weight varied by ethnicity and neighborhood deprivation, with benefits observed primarily among White British women, while no effect was found for Pakistani-origin participants. Conversely, studies in Barcelona found that living near major green spaces improved birth weight primarily among women from lower socioeconomic strata, while no effect was observed for higher SES groups (28).

Agay-Shay et al. (29) confirmed stronger associations between greenness and birth weight among women of lower SES in Israel, reinforcing the hypothesis that social vulnerability amplifies environmental risks. Reviews emphasize the need to integrate an environmental justice framework into urban planning and maternal health policies to address structural inequities in exposure to environmental hazards and access to protective factors such as green spaces (25, 55). Understanding these complex interactions is essential for designing interventions that reduce health disparities and maximize co-benefits for maternal and child health.

Beyond their direct association with birth outcomes, green spaces provide important co-benefits by mitigating environmental stressors such as heat, noise, and air pollution, which are recognized contributors to adverse pregnancy outcomes. Urban vegetation reduces the urban heat island effect and improves air quality through pollutant filtration, while also lowering noise levels, mechanisms that may indirectly support maternal and neonatal health (56, 57). These benefits are particularly relevant in the context of climate change adaptation strategies, where increasing urban greenery is considered a cost-effective intervention to reduce heat-related morbidity and mortality (58, 59). Evidence from systematic reviews confirms that regions with more green space report lower rates of heat-related health problems and deaths compared with those with little greenery, highlighting the role of green infrastructure in enhancing resilience to climate stressors (59, 60). Integrating green infrastructure into urban planning could therefore yield synergistic health benefits, extending beyond perinatal outcomes to broader population health.

The unequal distribution of green spaces across socio-economic strata raises critical concerns about environmental justice. Evidence suggests that the benefits of residential greenness on birth outcomes are not uniform and may be amplified among socially vulnerable populations. Similarly, systematic reviews highlight that structural inequities in access to green space contribute to persistent health disparities, particularly in urban areas where marginalized communities face compounded exposures to environmental hazards such as air pollution and heat (61, 62). These findings underscore the need for urban planning strategies that prioritize equitable distribution of green infrastructure and integrate community engagement to ensure accessibility and usability for all population groups (55, 63). Incorporating an environmental justice framework into maternal health policies is essential to address structural determinants of health and maximize co-benefits for maternal and child health.

The relevance of green space exposure extends beyond high-income countries. In low- and middle-income settings, where baseline exposure to environmental hazards such as air pollution and heat is higher and access to healthcare is limited, the potential benefits of urban greenery may be even more critical. However, evidence from these regions remains scarce, reflecting a gap in global research that limits the generalizability of current findings (12, 58). Expanding studies to diverse geographic and socio-economic contexts is essential to understand how green infrastructure can mitigate health risks and support climate adaptation strategies in vulnerable populations. Urban greening not only promotes maternal and child health but also contributes to reducing heat-related morbidity and mortality, improving air quality, and enhancing resilience to climate change (59, 60). These co-benefits highlight the importance of integrating green space interventions into sustainable development agendas and maternal health policies worldwide.

Our results confirm the positive effects of greenness on reproductive outcomes and, although the objective of this study was not to estimate disease burden, the process of standardization and reanalysis provides a robust methodological foundation for future applications in HIA. Harmonizing exposure metrics—such as transforming effect estimates to increments of 0.1 NDVI units—reduces heterogeneity and improves comparability across studies, which is essential for deriving reliable estimates for policy modeling. This approach was inspired by methodological principles proposed by Forastiere et al. (39) for the selection and validation of exposure–response estimates in environmental health assessments, adapted here to the context of green space exposure. However, our evaluation using AMSTAR 2 and AMSTAR2-EH led to overall lower quality ratings than those reported in the umbrella review by Forastiere et al. (39). This discrepancy does not reflect inconsistencies in the application of the tool, but rather differences in (i) the set of reviews included, (ii) the objective of the assessment, and (iii) the interpretation of AMSTAR2-EH critical items. First, Forastiere et al. (39) evaluated reviews covering a broader set of environmental exposures, many of which relied on standardized exposure metrics with well-defined transformation procedures. In contrast, most reviews included in our umbrella review combined heterogeneous greenness metrics (e.g., NDVI, proximity indices, green space percentage), often without providing sufficient information to allow harmonization into a common unit, which AMSTAR2-EH explicitly requires for environmental epidemiology. Second, our assessment focused specifically on the suitability of reviews for deriving exposure–response functions, a purpose for which AMSTAR2-EH places more stringent requirements on transparency, risk-of-bias evaluation, justification of excluded studies, and harmonization of effect estimates. Finally, several reviews in our set did not report excluded studies or did not transform their estimates into standardized units, leading to a higher number of unmet critical items. These structural differences explain why our ratings are more conservative and reflect the methodological challenges specific to the greenness–birth outcomes literature, thus supporting the need to conduct a new meta-analysis. In general, future assessments should prioritize studies with adequate methodological quality, transparent risk-of-bias evaluation, and harmonizable exposure metrics to ensure epidemiological validity. Moreover, while NDVI remains the most widely used proxy for greenness, it fails to capture critical attributes such as quality, accessibility, and actual use of green spaces, limiting causal inference (64). Integrating multidimensional indicators and leveraging high-resolution remote sensing data, combined with longitudinal designs and multi-exposure frameworks, will be crucial to disentangle complex interactions between environmental and social determinants of health.

A very recent umbrella review by Wang et al. (65) examined the relationship between greenness and a wide range of health outcomes, including pregnancy outcomes, reporting generally protective associations for birth weight and LBW and heterogenous findings for PTB. This review was published after we had completed our literature search and therefore could not be considered in our screening process. While Wang et al. (65) provide a valuable broad synthesis across multiple health domains and exposure metrics, their scope and methodological approach differ from ours. Our umbrella review focuses specifically on reproductive outcomes (9 systematic reviews vs. 5 selected in Wang, over the same period 2020–2024) and applies both AMSTAR 2 and AMSTAR2-EH to assess the suitability of existing reviews for generating exposure-response estimates. In addition, our work conducted a new harmonized meta-analysis of original studies by standardizing NDVI-based effect estimates and differentiating buffer distances and offers complementary insights that are particularly relevant for epidemiological interpretation and potential applications in HIA.

Recent large-scale study published after the completion of the literature search further support the robustness of the observed association between residential greenness and LBW (66). We therefore use this study to reinforce the interpretation and external validity of our findings.

This umbrella review has several strengths. It applies a rigorous methodological framework, including standardized exposure metrics and a harmonized meta-analysis, which reduces heterogeneity and improves comparability across studies. Unlike previous reviews, this approach provides discrete and robust estimates that can inform health policy and urban planning. However, some limitations should also be acknowledged. First, the evidence base relies predominantly on observational studies, which are susceptible to residual confounding and bias. Second, exposure assessment was largely based on NDVI, a proxy that does not capture critical dimensions such as quality, accessibility, and actual use of green spaces. Third, most primary studies were conducted in high-income countries, limiting the generalizability of findings to low- and middle-income settings where environmental risks and social vulnerabilities may differ substantially. Finally, high heterogeneity in study design and adjustment strategies underscores the need for more standardized protocols. Future research should prioritize longitudinal designs, integrate multidimensional indicators of greenness, and adopt multi-exposure frameworks to explore interactions with air pollution, heat, and noise.

In summary, this systematic review and harmonized reanalysis of available studies provide the most robust estimate to date for the protective effect of residential greenness on low birth weight (LBW), identifying the 300-meter buffer as the most reliable exposure metric for use in HIA. These findings are consistent with recent methodological guidance emphasizing hypothesis-driven buffer selection and the need to test multiple scales to improve comparability and reduce bias (51). However, the findings underscore that the benefits of green space, though universally generalizable, require land-use and urban planning policies to integrate the principle of environmental justice in a case-by-case base.

## Data Availability

Publicly available datasets were analyzed in this study. This data can be found here: the data used in this study were extracted from previously published primary studies included in the systematic reviews and meta-analyses. No new datasets were generated or deposited, and all extracted information is available within the original publications as cited in the article.

## Footnotes

1 Remote sensing method that uses pulsed laser light to measure variable distances to the Earth or objects in the environment.
