# Supplementary material for: Residential greenness and birth outcomes: evidence for reduced low birth weight from an umbrella review and harmonized normalized difference vegetation index synthesis
Source: Front Public Health. 2026 Jun 18;14:1816767. doi: 10.3389/fpubh.2026.1816767 (PMC13323503; doi:10.3389/fpubh.2026.1816767)
Supplement: Supplementary file 1 [file Table_1.docx]

Table S1. Search strategy.

| **Data Sources** | **Search Strategy** |
| --- | --- |
| PUBMED | ("Pre term birth"[tiab:~0] or "Pre term births"[tiab:~0] or "Pre term infant"[tiab:~0] or "Pre term infants"[tiab:~0] or "premature birth*" [tiab] or "premature infant*"[tiab] or "neonatal prematur*"[tiab] or "Premature labor"[tiab:~1] or "Pre term labor"[tiab:~0] or "Premature labour"[tiab:~1] or "Pre term labour"[tiab:~0] "low birth weight"[tiab:~1] or "premature birth"[mh] or infant, premature[mh] or infant, low birth weight [tiab] Or preterm birth [tiab] or preterm infant[tiab] or preterm outcomes[tiab] OR preterm outcome[tiab] OR pregnancy outcome*[tiab] OR birth outcome*[tiab] OR "small for gestational age"[tiab:~0] OR SGA[tiab] OR "Sex ratio"[mh] or sex ratio[tiab]) AND (Green area*[Title/Abstract] OR green space*[Title/Abstract] OR greenness[Title/Abstract] OR greening[Title/Abstract] OR "residential green"[tiab] OR "community park"[tiab] OR "national park"[tiab] OR "urban park"[tiab] OR "recreational park"[tiab] OR "community parks"[tiab] OR "national parks"[tiab] OR "urban parks »[tiab] OR "recreational parks"[tiab] OR "parks, recreational"[MeSH Terms]) Filters: Systematic Review, English, Italian, from 2020 - 3000/12/12 |
| EMBASE | ('pre term birth:ti,ab' OR 'pre term births:ti,ab' OR 'pre term infant:ti,ab' OR 'pre term infants:ti,ab' OR 'premature birth*':ti,ab OR 'premature infant*':ti,ab OR 'neonatal prematur*':ti,ab OR 'premature labor:ti,ab' OR 'pre term labor:ti,ab' OR 'premature labour:ti,ab' OR 'pre term labour:ti,ab' OR 'low birth weight:ti,ab' OR 'premature birth'/exp OR 'infant, premature'/exp OR 'infant, low birth weight':ti,ab OR 'preterm birth':ti,ab OR 'preterm infant':ti,ab OR 'preterm outcomes':ti,ab OR 'preterm outcome':ti,ab OR 'pregnancy outcome*':ti,ab OR 'birth outcome*':ti,ab OR 'small for gestational age:ti,ab' OR sga:ti,ab OR 'sex ratio'/exp OR 'sex ratio':ti,ab) AND ('green area*':ti,ab OR 'green space*':ti,ab OR greenness:ti,ab OR greening:ti,ab OR 'residential green':ti,ab OR 'community park':ti,ab OR 'national park':ti,ab OR 'urban park':ti,ab OR 'recreational park':ti,ab OR 'community parks':ti,ab OR 'national parks':ti,ab OR 'urban parks':ti,ab OR 'recreational parks':ti,ab OR 'parks, recreational'/exp) AND 'systematic review'/de AND ([english]/lim OR [italian]/lim) |
| SCOPUS | (TITLE-ABS("pre term birth") OR TITLE-ABS("pre term births") OR TITLE-ABS("pre term infant") OR TITLE-ABS("pre term infants") OR TITLE-ABS("premature birth*") OR TITLE-ABS("premature infant*") OR TITLE-ABS("neonatal prematur*") OR TITLE-ABS("premature labor") OR TITLE-ABS("pre term labor") OR TITLE-ABS("premature labour") OR TITLE-ABS("pre term labour") OR TITLE-ABS("low birth weight") OR TITLE-ABS("premature birth") OR TITLE-ABS("infant, premature") OR TITLE-ABS("infant, low birth weight") OR TITLE-ABS("preterm birth") OR TITLE-ABS("preterm infant") OR TITLE-ABS("preterm outcomes") OR TITLE-ABS("preterm outcome") OR TITLE-ABS("pregnancy outcome*") OR TITLE-ABS("birth outcome*") OR TITLE-ABS("small for gestational age") OR TITLE-ABS("SGA") OR TITLE-ABS("sex ratio")) AND (TITLE-ABS("green area*") OR TITLE-ABS("green space*") OR TITLE-ABS("greenness") OR TITLE-ABS("greening") OR TITLE-ABS("residential green") OR TITLE-ABS("community park") OR TITLE-ABS("national park") OR TITLE-ABS("urban park") OR TITLE-ABS("recreational park") OR TITLE-ABS("community parks") OR TITLE-ABS("national parks") OR TITLE-ABS("urban parks") OR TITLE-ABS("recreational parks") OR TITLE-ABS("parks, recreational")) AND ( LIMIT-TO ( DOCTYPE,"re" ) ) AND ( LIMIT-TO ( LANGUAGE,"English" ) OR LIMIT-TO ( LANGUAGE,"Italian" ) ) |
