# Supplementary material for: Residential greenness and birth outcomes: evidence for reduced low birth weight from an umbrella review and harmonized normalized difference vegetation index synthesis
Source: Front Public Health. 2026 Jun 18;14:1816767. doi: 10.3389/fpubh.2026.1816767 (PMC13323503; doi:10.3389/fpubh.2026.1816767)
Supplement: Supplementary file 2 [file Table_2.docx]

Table S2 - Socio-geographical context and environmental settings of the included systematic reviews.

| **Review (Author, Year)** | **Geographic Coverage** | **Predominant Setting** | **Population/Context Details** |
| --- | --- | --- | --- |
| Ahmer (2024) | Global | Urban / Mixed | Includes studies from North America, Europe, China, and Oceania. Considers socio-economic factors and urban heat island effects as potential confounders. |
| Rigolon (2021) | Global | Urban / Mixed | Focus on health inequities and environmental justice in urban areas. |
| Hu (2021) | Global | Urban / Mixed | High representation of Asian (China) and North American cohorts. |
| Mygind (2021) | Global | Urban / Mixed | Primarily high-income countries (HICs) with diverse urban vegetation. |
| Lee (2020) | Global | Urban / Mixed | Multi-continental coverage including high-density urban settings. |
| Akaraci (2020) | Global | Urban / Mixed | Comprehensive global synthesis across different climate zones. Discusses different climate zones and seasonal variations. |
| Zhan (2020) | Global | Urban / Mixed | Strong focus on metropolitan areas and satellite-based NDVI metrics. |
| Islam (2020) | Global | Urban / Mixed | General population across diverse geographical regions. |
| Siddika (2023) | Global | Urban / Mixed | Global evidence focusing on air pollution and greenness interaction. Evaluates interaction with air pollution and temperature-related factors. |
